# Spectrophotometric and chromatographic strategies for exploring of the nanostructure pharmaceutical formulations which contains testosterone undecanoate

**DOI:** 10.1038/s41598-020-60657-4

**Published:** 2020-02-27

**Authors:** Monica Butnariu, Ioan Sarac, Ionel Samfira

**Affiliations:** Banat’s University of Agricultural Sciences and Veterinary Medicine “King Michael I of Romania” from Timisoara, 300645, Calea Aradului 119, Timisoara, Timis Romania

**Keywords:** Analytical biochemistry, Assay systems, Bioanalytical chemistry

## Abstract

The nanostructure pharmaceutical formulations (NPF) which contains testosterone undecanoate (TU) been used in life science as parent compound delivery systems for therapeutic, but and been used to enhance the performance in sport, so it is a significant substance for public health and nutritional supplements. In most Pharmacopoeias testosterone is described as an ester of some lower acids (often propionate). The aim of this study is to determine purity of the parent compound by chromatography and absorption spectrum in the frame of middle infrared. Chemical structure of undecanoate was prepared and used in order to achieve a better absorption. This is explained by increased lipophilicity of undecanoate. Due to its lipophilic character, TU is soluble in non–polar solvents but shows a satisfactory solubility in absolute ethanol. Based on the molecular structure, a moderate absorption in the frame of UV with a maximum absorption at a not too high wave-length can be predicted. Maximum absorption occurs in a spectral region in which usual ingredients do not present significant interference.

## Introduction

The importance of analytical techniques used in the detection and determination of drugs, their impurities and degradation products, play an important role in human health^1^.^,^ HPLC has been cited extensively in the analysis of pharmaceutical compounds in the literature. The advantage of HPLC is its ability to have reproducible and reliable analysis and has the advantage in its reliance on the solubility of the analyte in contrast with the analyte volatility (for GC analysis). HPLC also offers useful detection systems; the UV detector is on the whole inexpensive when versus with the mass spectrometric detector. UV detection is adequate for routine analysis and is well suited for purity many pharmaceutical compounds, which have UV chromophores^2^. The specificity of the HPLC technique and infrared absorption spectra procedure, is very good and simultaneously sufficient precision is also attainable, for determine purity of the parent compound^3^.

NPF by the anabolic androgenic steroids (AAS) chemically produced are structurally related with the testosterone parent compound. The latter were adapted to improve its the pharmacological properties and anabolic effect of the testosterone (androst–4–ep–17–ol–30) over the protein, while in the same time reducing unwanted androgenic effects^4^. NPF like AAS can be divided into the following the groups: analogues obtained by esterification of the 17–β–hydroxyl group; analogues alkylated in 17–α position; analogues with the modified nuclei A, B and C^5^. Esterification of the 17–β–hydroxyl group with the carboxylic acids enables intramuscular administration of substance^6^. The most common 17–β esters are derived from the natural testosterone (i.e. testosterone cypionate, propionate, enanthate and undecanoate). Alkylation of 17α position protects metabolic steroids to the first metabolic phase (“first pass metabolism”) allowing oral administration^7^. Most 17–alkylated AAS are methyl derived, but also there are ethylic and ethinyl derivated. Most of the ASA present structural modifications in the nuclei A, B or C^8^. This type of steroid is often available for oral use, therefore relevant to human nutrition aspects^9^.

**Figure 1:**
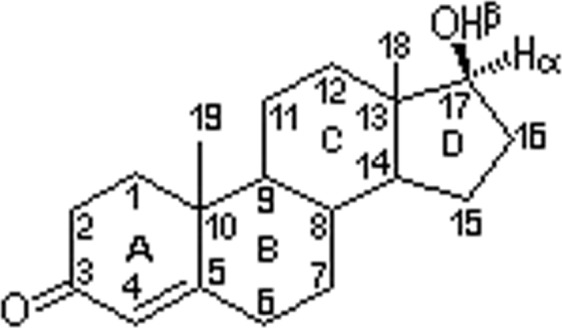
Chemical structure of the testosterone.

It is clear that the most common changes include the introduction of double bonds between C1 and C2 atoms and reduction of the double bond between C4 and C5 atoms. Other modifications include: methyl group attachment to C1 atom; methyl or oxymethyl group attachment to the C2 or its replacement with oxygen; attachment of the pyrazole or other cyclic structure to nucleus A through the C2 and C3; attachment of a chlorine atom or hydroxyl group to C4; attachment of a methyl group to C7. Nandrolone, the most common analog testosterone (Fig. 1) shows the structure of testosterone, but the angular methyl group C19 between the nuclei A and B is absent. In many countries besides the synthetic NPF like AAS there are a lot of other steroids which are commercialized as “dietetic supplements” or “prohormones”^10,11^. Dehydroepiandrosterone (DHEA) and 4-androstenedione are the androgenic steroids. Other chemical substances related to the later are 5-androstenedione, 4-androstenediol, 5-androstenediol, 19-norandrosta-4-enedion, 19-norandrosta-5-enediol and 19-norandrosta-4-enediol. Most of those steroids have no any known clinical recommendation^12^. A new trend in the use of AAS is represented by the designer steroids that are not commercially available. Such steroids are designed to be undetectable in tests^13^.

The first agent from this category found and used was norbolethone. Later, two other steroids were developed, tetrahydrogestrinone (THG) and Madol (17α-methyl-5α-androst-2-en-17β-ol). Like many other the prohormones their efficiency and toxicity are unclear^14^. The metabolism of ASA compounds is internal and follows that of testosterone and other steroids^15^. The metabolic route includes oxidation, reduction, hydroxylation and epimerization reactions (Phase I) and conjugation reactions forming glucuronides and sulphate conjugates (Phase II reactions)^16,17^.

Doping with the testosterone and many other natural steroids can be directly confirmed by mass spectrometry coupled with combustion and carbon isotope ratio determination mass spectrometry coupled with combustion and carbon isotope ratio determination^18^. This method is based on the finding that the endogenous steroids and those obtained by chemically show little difference (but detectable) of the carbon isotope ratio of (^13^C/^12^C). Chemically obtained steroids are synthesized from certain plant sterols with a low level of ^13^C, while the content of ^13^C in the human body is higher and reflects the person’s diet^19^. During analyze the ^13^C/^12^C ratio for potential administered steroids or their metabolites (androsterone, etiocholanolone, 5α-androstane-3α, 17β-diol, 5β-androstane-3α, 17β-diol) is determinate and compared the ^13^C/^12^C ratio of other endogenic steroids which are not affected by administered steroid (pregnanediol, 11–ketoetiocolonolon)^20^.

There are two types of analyses for ASA compounds and other substances: “screening” procedures and methods of analysis for confirmation. The aim of the “screening” is to discover samples to be analyzed. A perfect “screening” method must be simple, rapid, selective, sensitive, economic and not leading to unnecessary use of sample. Due to the large number of analytes and to minimize the number of procedures involved, multi–analytical detection methods are preferred. The development of the NPF brought a revelation in life science. These NPF would it helps their intent only if they are free from impurities. To realize parent compound, it is important to use different analytical chemical and instrumental methods, techniques was involved in the estimation of the parent compound. These NPF may develop impurities at various steps of their design, preparation, transportation and stowage which makes the life science risky to be administered thus they must be detected and quantitated. This research highlights the role of the analytical methods in assessing the quality of the parent compound, by using some techniques such as chromatographic, spectroscopic and spectrophotometric dosing, their corresponding methods that have been applied in the analysis of NPF.

## Materials and Methods

**Figure 2:**
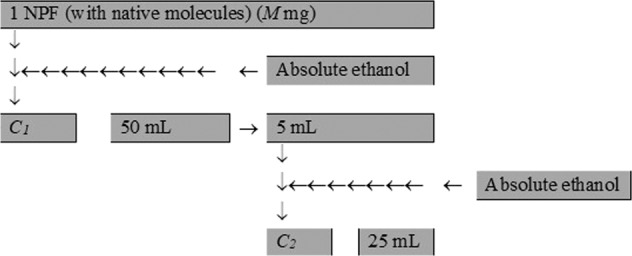
Mode of the quantitative determination.

The aim of this study was determining the purity (by various analytical methods) of a NPF, which contains TU. The solvents used in this work namely hexane, acetonitrile, ethanol, and acetic acid were purchased from Merck or Fluka and had a purity of >99% (vol /vol). Carrying out the method of analysis involves the following operations (Fig. 2): the contents of a NPF was discharged in a 50 mL volumetric flask, after dissolving in absolute ethanol; with the same solvent the contents of the volumetric flask is brought to the level; 5 mL of the obtained solution is diluted in a second 25 mL volumetric carboy.

The final solution was subjected to spectrophotometric measurement in a quartz cuvette, having a thickness of 0.5 cm. For preparation of calibration curve, the concentrations used ranged between 40–200 mg/L for each method.

The *infrared absorption spectra* were recorded with infrared spectroscopy (FTIR, model “460 Plus”, Jasco Products Co.). Samples were included in a potassium bromide (Technique KBr 10.65 mg, well 10 μg/mL) tablet (potassium bromide of spectral purity was used (“Uvasol”, Merck). Data processing was done with specialized “Jascow” software (Jasco Company). The sample concentration was 40,00 mg. TU in test sample was calculated in quantitative and percentage basis from measured peak area response for the test sample (Au), compared to standard peak area response (As) using following equations:

$${\rm{Quantity}}={\rm{Au}}/{\rm{As}}\times {\rm{C}}(1) \% \,{\rm{Recovery}}=({\rm{Observed}}\,{\rm{Amount}})/({\rm{Declared}}\,{\rm{Amount}})\times 100.$$

Where C is the concentration in ppm of the TU.

The *chromatography study* was made with a JASCO HPLC apparatus. For the study of chromatographic properties of the parent compound and possible impurities a reverse C18 (250 × 4.6 nm) stationary phase column was used. As mobile phase has been used absolute methanol purity, from MERCK. Flow passage of mobile phase through the column was 1 mL/min. Monitoring of the eluent was carried out at 240 nm, due to the fact that, in alcoholic environment at this wave-length the parent compound presents a maximum optical absorption. In the working conditions choosing the eluent was justified because the interest component showed a good retention (6.7 min.). Quantification were done with injection volume of 10 μL sample with concentration 40,00 mg. The experiments were carried out in triplicate^21^.

*Absorption spectra in the ultraviolet frame* (UV) were recorded with a double beam spectrophotometer, model PG Instruments UV–VIS spectrophotometer using the UV WIN 5.05 software. The samples were placed in quartz cuvettes with 0.5 cm length path. Weight was performed with a semi-micro analytical balance (resolution ± 0.1 mg) (Sartorius, model BSA224S-CW).

*Statistical testing* of the functional dependence was performed using IBM SPSS Statistics 23.

## Results and Discussion

### The absorption spectrum in the middle infrared frame (IR) study

IR absorption spectra are generated by the transitions between the states of vibration of the molecules from the sample used in research. In general, IR absorption spectrum of the TU film dried from the dichloromethane solution deposited onto the CaF_2_ surface.

**Figure 3:**
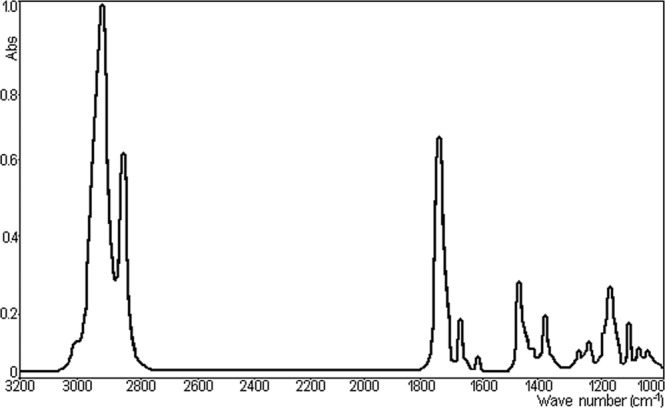
Absorption spectrum in the frame 3200–1000 cm^−^¹ of TU.

The typical peaks in the TU film IR spectrum shown in Fig. 3, that are presented the absorption spectrum of the frame of 3200–1000 cm^−^¹. Absorption bands in the infrared region identified in the Fig. 3 result from interatomic vibrations, whose frequencies are related to the strength of the atomic bonds involved. It should so be possible to assign certain absorption bands to particular atomic groups or linkages. They should be caused by the following vibrations: C–H aromatic ring deformations around 2950 cm^−1^; C=O carbonate group deformations near 1745 cm^−1^; C=C-vibrations at 1460 cm^−1^; asymmetric O–C–O carbonate group deformations in the range 1230–1150 cm^−1^; CH_3_-vibrations at 1050 cm^−1^; symmetric O–C–O carbonate group deformations near 1000 cm^−1^ ^22^.

Although the viscous consistency of the samples, in general, makes is *difficult* to *visualize* of the absorption bands because of intense intermolecular interactions, the spectrum obtained with advanced technique FT/IR convincingly shows some characteristic bands of the molecule. To this intense complexe bands between 2850 and 2950 cm^−^¹ symmetrical and asymmetrical vibrations of the simple C–H bond of the methyl (−CH_3_), methylene group or methylidene (=CH) and methine or methylylidene (≡C−) can be attributed. Strong band located at 1745 cm^−^¹ is assigned to the stretching vibration of the carbonyl group.

The positioning to the relatively small number of wave-length of this bands can be explained by the conjugation of the groups with C=C by a double bond located in the neighborhood (vicinity). The strip of bands located between 1375–1460 cm^−^¹ is characteristic to the deformations vibrations of methyl groups, methylene and methyl. Relatively wide band and medium intensity lying between 1175 and 1200 cm^−^¹ can be attributed to the vibration of simple bonds C–C in the molecular skeleton.

### The chromatographic study

**Figure 4:**
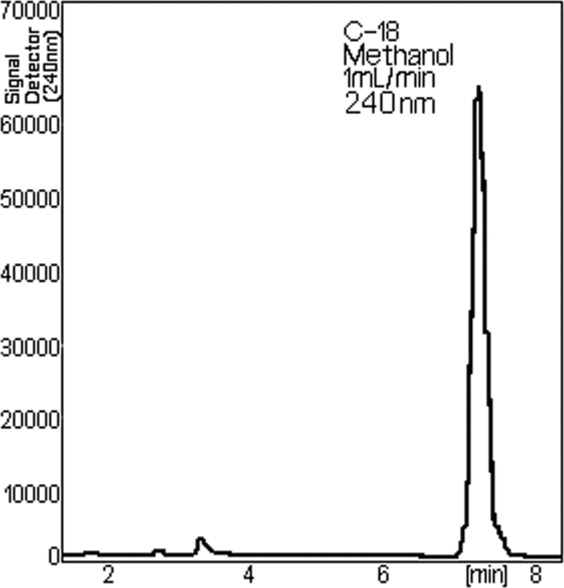
Chromatogram of the TU sample.

The chromatography is a method of separating many different of chemical mixtures. In the Fig. 4 is represented the chromatogram of the sample which was analysed, the content of NPF. It is noted the symmetrical shape of the dominant peak, proving that the working conditions were satisfied. Besides the parent compound, due to the accompanying impurities which join the major component minor peaks were registered.

**Figure 5:**
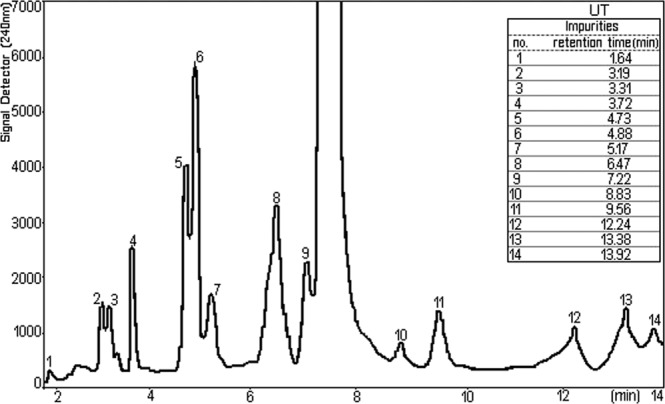
Chromatogram of NPF1 with impurities, for the sample (peaks 1–14 represent impurities).

**Figure 6:**
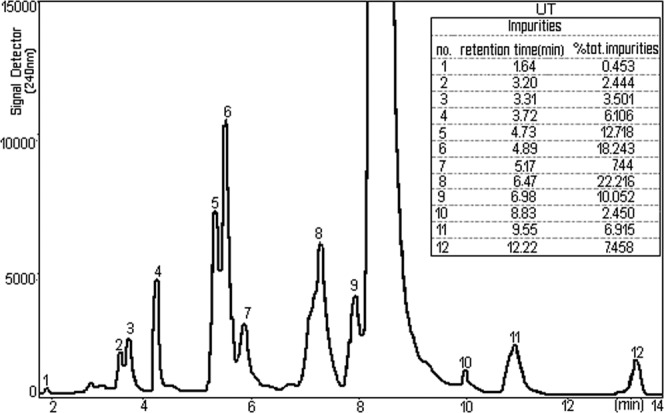
Chromatogram of NPF2 with impurities, for the sample (peaks 1–12 represent impurities).

Figures 5 and 6 represent chromatograms of the content of different NPF with advanced extension of the ordinate axis, signal detector. It appears that the interest component, TU is accompanied by 14 or 12 impurities^23^.

While the chromatograms shown in Fig. 5 (peaks 1–14 represent impurities) and 6 (peaks 1–12 represent impurities) were processed from different NPF, general aspect of the peaks attributed to impurities is basically the same except that, in the case of the chromatogram represented in Fig. 6 do not show the impurities of the number 13 and 14 presented in the Fig. 5.

Each chromatographic peak represents a separate impurity. If we refer to the total amount of impurities, we look Fig. 6, assuming 100% (wt/vol) the presence of individual impurities range between 0.453 and 22.216% (wt/vol). The isolations that are used for chemical analysis in HPLC are carried out by injecting a of sample onto the chromatographic system. So, results in a HPLC chromatogram that compiles of a sequence of peaks that represent the different impurities and parent compound (TU) in the NPF sample as they each elute from the column. The retention time or retention volume of each peak can be used to support recognize the eluting impurities and TU, whereas the area or height of the peak can be used to measure the amount of the impurities and TU that is present. The width of each peak is also of precious in a chromatogram. The peak width depicts the separating performance or efficiency of the HPLC system.

### The spectrophotometric TU study

Due to the presence of a conjugated double bond C=O with double bond C=C in the molecule, the compound exhibits a maximum optical absorption between 220–240 nm. It notes that the exact position of maximum absorption is dependent, in a significant manner by the properties of the solvent in which the absorption spectrum was recorded^24^.

**Figure 7:**
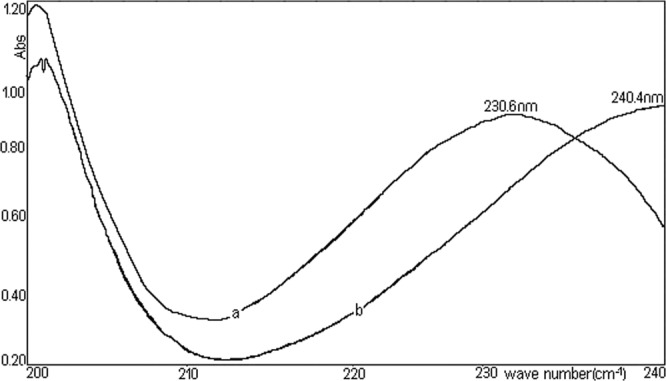
Absorption spectra of TU in hexane [**b**] and absolute ethanol [**a**].

Figure 7 shows absorption spectra corresponding to two extreme situations: using hexane and absolute ethanol as solvents. It is noted that increasing the polarity causes a marked bathochromic movement of the maximum absorption (about 10 nm, shows a bathochromic stretch of the absorption maximum upon increasing the solvent polarity). The accentuated dependence of the maximum position of absorption to the solvent polarity supports the supposition that the maximum absorption is a result of the electronic transition of the type n–π*. Absorption maxima of TU are influenced by with solvent polarity. Solvation of TU molecules occurs via dipole-dipole interactions in non-H-bond donating solvents, inasmuch as in H-bond donating solvents the occurrence is more H-bonding^25,26^.

**Figure 8:**
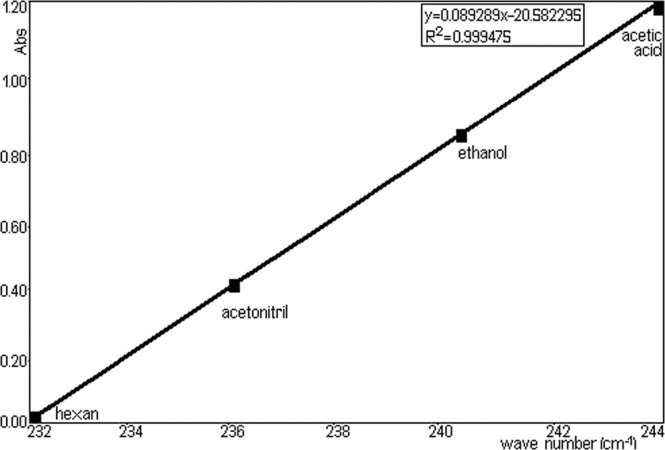
Linear dependence of the maximum position of absorption parameter.

**Figure 9:**
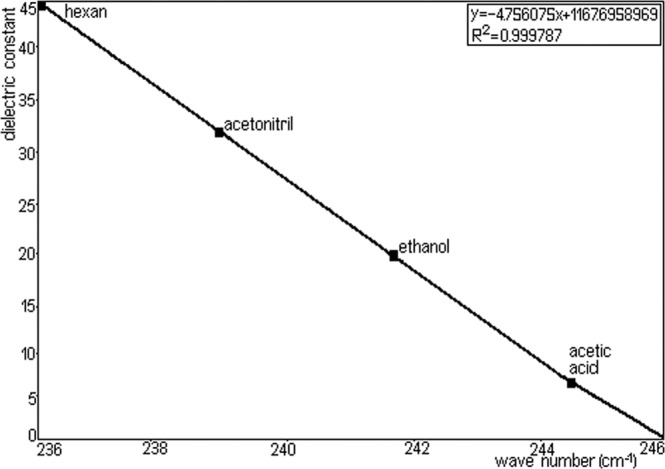
Linear dependence of the maximum position of absorption for dielectric constant solvent (relative permittivity).

A Figs. 8 and 9 represents the surprisingly linear dependence of the position of absorption m aximum from the dielectric constant of the solvents: hexane (1.89), acetonitrile (36.64), ethanol (24.6), acetic acid (6.20). Spectral solvents must be transparent in the domain of work. In the visible domain one can work, practically, with any colorless solvent, the most common solvents are compounds containing only H.

Solvents should be of high purity in the sense that they do not have to contain impurities, absorbing in spectral region. Absorption stretch of TU solution as a function of solvent polarizability (π*), from Fig. 8 (y = −0.089289x + 20.582295) shows that the wave number (wave-length) in the maximum of π-π* absorption band of TU depends linearly on the dielectric function of solvents. So, a good correlation (R^2^ = 0.999475) is seen between dielectric function of solvents (hexane, acetonitrile, ethanol and acetic acid).

H-bonding acceptor capacity of the solvents, does not correlate with the wave-length in the maximum of the π-π*. For TU the absorption spectroscopy deal with the transition of an electron from bonding (π, σ and n) to antibonding orbital (π* and σ*). The σ → σ* transition requires energy which does not fall in the UV-Vis range (only π → π* and n chemical group π* transitions occur in the UV-V is region, but the energy gap between π and π* is higher than that of between n and π*. Therefore, n → π* transitions are more favourable than that of π→π* transitions. Saturated compounds containing unshared electron pairs (lone electron carriers) show n-π* transitions. The transitions can be related to the absorption properties of some chemical group present in the molecule (chromophore absorbs UV-V radiation at a specific wave-length, with little influence from the other groups in the compound molecule).

TU has typical chromophores such as C=C double bonds, C=O carboxylic groups and aromatic rings. The chemical group as chromophores interact with the solvent and with other chromophores and modify position, intensity, and shape of absorption bands. So, we tried to analyse the solvents effects on the TU using π-π* absorption band. It was obtained that some solvents suggested a good linearity for H bond acceptor basicity parameter, dielectric constant with respect to wave-length. This showed that the H bond acceptor basicity parameter is the significant parameter for both dissociation and complex formation reactions, and the H bonding acceptor capacity and the induction-dispersive forces of solvent molecules have caused the bathochromic stretch in absorption maxima of TU. A sketching of λ_max_ versus the absorption parameter values in various solvents is shown in Fig. 8 (y= − 0.089289x + 20.582295, R^2^ = 0.999475).

From Fig. 9 of λ_max_ versus the dielectric constant values (y= − 4.756075x + 1167.6958969, R^2^ = 0.999787), it can be seen that with increasing dielectric constant values, the spectrum is stretched to higher wave-length. An increase in λ_max_ values with π* (dipolarity/polarizability) also indicates that TU interaction becomes different with increasing capability of a given solvent to form H bonds in solution. In order to develop a method for quantitative determination of TU in the NPF the compliance quantitative Lambert–Beer relationship in the concentration range 20–250 mg/L was tested (in this the concentration range, was obtained the linearity of method). As solvent was chosen absolute ethanol by spectroscopic purity (Uvasol–Merck).

**Figure 10:**
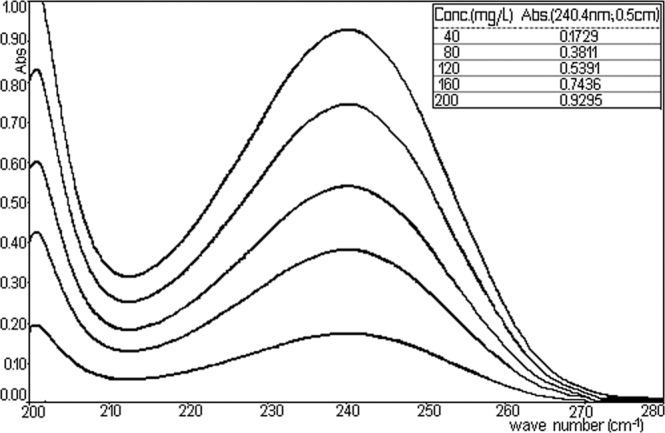
Absorption spectra of TU (in absolute ethanol) spectra for calibration.

Figure 10 represents the observation overlap spectra of the five ethanol solutions containing TU ranged between 40–200 mg/L.

**Figure 11:**
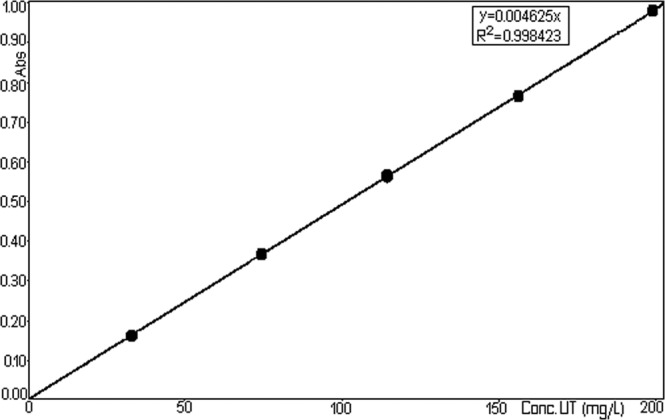
Right calibration of TU.

Absorbance values read at a 240.4 nm wave-length in a cuvette with 0.5 cm thickness are plotted against the concentration of TU in Fig. 11 (Right calibration of TU).

It is noted an almost perfect linearity between these two sizes ensuring the applicability of Lambert–Beer relationship in the mentioned concentration range.

To mathematically describe the strength of correlation between the two variables, was obtained the equation y = 0.004625x, with the correlation coefficient R^2^ = 0.998423 (Fig. 11).

**Figure 12:**
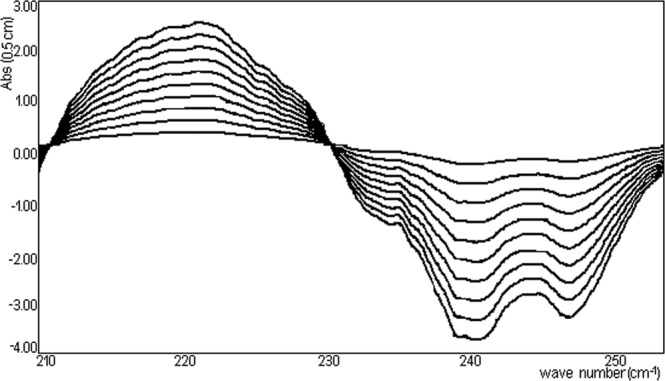
Spectra absorption of TU (in hexane).

Figure 12 represents the family of curves obtained by deriving the absorbance upon wave-length for a series of ten reference solutions with a concentration of the compound of interest between 25–250 mg/L.

**Figure 13:**
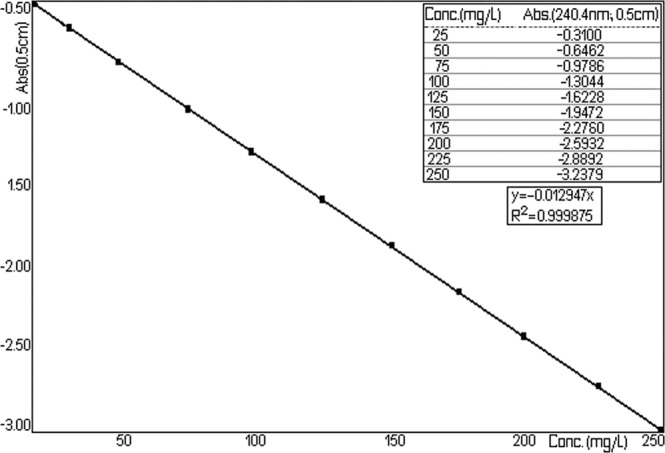
Concentration value linearly read at 247 nm versus absorbance.

The value read at 247 nm is linearly correlated with the concentration level according Fig. 13 (y = −0.012947, R^2^ = 0.999875) for a series of ten reference solutions with a concentration of the compound of interest between 25–250 mg/L. Checking of Beer–Lambert relationship in the concentration domain, it enables the development of an analytical method for the determination of TU from the NPF^27,28^.

Based on absorbance readings at 240.4 nm of samples diluted in absolute ethanol the analysis method has been developed and tested. Table 1 provides results in 10 samples NPF from the same sample. The absorption values are read at 240.4 nm.

**Table 1:** Provides the results of 10 NPF from the same sample. The absorption values are read at 240.4 nm.

A (absolute ethanol) (240.4 nm; 0,5 cm)	*C*_2_(mg/L)	*C*_1_ (mg/L)	*M* (mg/caps.)
0.7466	161.4	807.00	40.350
0.7502	162.2	811.00	40.550
0.7433	160.7	803.50	40.175
0.7415	160.3	801.50	40.075
0.7517	162.5	812.50	40.625
0.7508	162.3	811.50	40.575
0.7483	161.8	809.00	40.450
0.7388	159.7	798.50	39.925
0.7378	159.5	797.50	39.875
0.7405	160.1	800.50	40.025
		Average **(mg)**	**40.262**
		Standard deviation **(mg)**	**0.705**

The last column of Table 1 contains the amount of TU/sample (between 40.625 mg–39.875 mg). Average the 10 values is 40.242 mg (closest to the declared value 40 mg) and the standard deviation around the mean of the individual values is from 0.705 mg, i.e. 1.75% (wt/vol) of the declared value. It shows that the standard deviation falls within limits of European pharmacopoeia individual deviations from the declared value. The last column of Table 1 contains the amount of TU/sample (ranged between 40.625 mg–39.875 mg). The average of the 10 values is 40.242 mg (close to the declared value of 40 mg) and the standard deviation of the individual values around the average is 0.705 mg i.e. 1.75% (wt/vol) of the declared value. The statistical study on the functional dependence of the of the method used in this article compared to other methods, indicated the logarithmic function of the form A = b_0_ + b_1_ln(M) as a model that objectively describes their relation. Specifically, the expression of the function is A = − 2.001236 + 0.743137 ln(M). Regarding the testing of the coefficients, the values t = 205.6 were obtained for the coefficient of ln (M) respectively t = −149.8 for constant, both situations indicating a high statistical significance, sig. <0.001. And the value F = 42296.2, corresponding to the ANOVA table, has statistical significance, sig. <0.001 (Supplementary Information)^29^. Concerning individual deviations from the declared value, the standard deviation falls within the limits of European pharmacopoeia.

## Conclusions

The maximum absorption of the substance is dependent in a significant manner by the properties of the solvent in which the absorption spectrum was recorded. Maximum absorption is due to electronic transitions of the type n–π*. Electronic absorption spectra showed that solvent polarity effects π-π∗ band of TU. The analysis method developed and tested demonstrates that the maximum absorption depends on the dielectric constant of the solvents (hexane, acetonitrile, ethanol and acetic acid) respectively. The linearity between these two sizes (absorbance values read at a 240.4 nm wave-length in a cuvette with a thickness of 0.5 cm, plotted against the concentration of TU) ensure the applicability of the Lambert–Beer relationship in the mentioned concentration. Checking the Lambert–Beer relationship, in particular concentration, allowed the development of an analytical method for the determination of TU from the NPF. Standard deviation falls in the limits allowed by the European pharmacopoeia concerning individual deviations from the declared value.

## Supplementary information

Supplementary Information.
