# Supplementary material for: Spectrophotometric and chromatographic strategies for exploring of the nanostructure pharmaceutical formulations which contains testosterone undecanoate
Source: Sci Rep. 2020 Feb 27;10:3569. doi: 10.1038/s41598-020-60657-4 (PMC7046639; doi:10.1038/s41598-020-60657-4)

The statistical study on the functional dependence of the of the method used in this article compared to other methods, indicated the logarithmic function of the form A=b_0_+b_1_ln(M) as a model that objectively describes their relation. Specifically, the expression of the function is A =– 2.001236 + 0.743137 ln(M). Regarding the testing of the coefficients, the values t = 205.6 were obtained for the coefficient of ln (M) respectively t = –149.8 for constant, both situations indicating a high statistical significance, sig. <0.001. And the value F = 42296.2, corresponding to the ANOVA table, has statistical significance, sig. <0.001.

* Curve Estimation.

TSET NEWVAR=NONE.

CURVEFIT

/VARIABLES=A WITH M

/CONSTANT

/MODEL=LINEAR

/PRINT ANOVA

/PLOT FIT.

**Curve Fit**

| **Notes** | | | | | | | | |
| --- | --- | --- | --- | --- | --- | --- | --- | --- |
| Output Created | | | | | 11-DEC-2019 14:38:43 | | | |
| Comments | | | | |  | | | |
| Input | | Data | | | F:\fe.sav | | | |
|  |  | Active Dataset | | | DataSet1 | | | |
|  |  | Filter | | | <none> | | | |
|  |  | Weight | | | <none> | | | |
|  |  | Split File | | | <none> | | | |
|  |  | N of Rows in Working Data File | | | 10 | | | |
| Missing Value Handling | | Definition of Missing | | | User-defined missing values are treated as missing. | | | |
|  |  | Cases Used | | | Cases with a missing value in any variable are not used in the analysis. | | | |
| Syntax | | | | | CURVEFIT  /VARIABLES=A WITH M  /CONSTANT  /MODEL=LINEAR  /PRINT ANOVA  /PLOT FIT. | | | |
| Resources | | Processor Time | | | 00:00:00.14 | | | |
|  |  | Elapsed Time | | | 00:00:00.13 | | | |
| Use | | From | | | First observation | | | |
|  |  | To | | | Last observation | | | |
| Predict | | From | | | First Observation following the use period | | | |
|  |  | To | | | Last observation | | | |
| Time Series Settings (TSET) | | Amount of Output | | | PRINT = DEFAULT | | | |
|  |  | Saving New Variables | | | NEWVAR = NONE | | | |
|  |  | Maximum Number of Lags in Autocorrelation or Partial Autocorrelation Plots | | | MXAUTO = 16 | | | |
|  |  | Maximum Number of Lags Per Cross-Correlation Plots | | | MXCROSS = 7 | | | |
|  |  | Maximum Number of New Variables Generated Per Procedure | | | MXNEWVAR = 60 | | | |
|  |  | Maximum Number of New Cases Per Procedure | | | MXPREDICT = 1000 | | | |
|  |  | Treatment of User-Missing Values | | | MISSING = EXCLUDE | | | |
|  |  | Confidence Interval Percentage Value | | | CIN = 95 | | | |
|  |  | Tolerance for Entering Variables in Regression Equations | | | TOLER = .0001 | | | |
|  |  | Maximum Iterative Parameter Change | | | CNVERGE = .001 | | | |
|  |  | Method of Calculating Std. Errors for Autocorrelations | | | ACFSE = IND | | | |
|  |  | Length of Seasonal Period | | | Unspecified | | | |
|  |  | Variable Whose Values Label Observations in Plots | | | Unspecified | | | |
|  |  | Equations Include | | | CONSTANT | | | |
| **Model Description** | | | | | | | |  |
| Model Name | | | | | MOD_8 | | |  |
| Dependent Variable | | 1 | | | A | | |  |
| Equation | | 1 | | | Linear | | |  |
| Independent Variable | | | | | M | | |  |
| Constant | | | | | Included | | |  |
| Variable Whose Values Label Observations in Plots | | | | | Unspecified | | |  |
| **Case Processing Summary** | | |  |  |  |  |  |  |
|  | N | |  |  |  |  |  |  |
| Total Cases | 10 | |  |  |  |  |  |  |
| Excluded Cases^a^ | 0 | |  |  |  |  |  |  |
| Forecasted Cases | 0 | |  |  |  |  |  |  |
| Newly Created Cases | 0 | |  |  |  |  |  |  |
| a. Cases with a missing value in any variable are excluded from the analysis. | | |  |  |  |  |  |  |
| **Variable Processing Summary** | | | | | | |  |  |
|  | | | | Variables | | |  |  |
|  |  |  |  | Dependent | | Independent |  |  |
|  |  |  |  | A | | M |  |  |
| Number of Positive Values | | | | 10 | | 10 |  |  |
| Number of Zeros | | | | 0 | | 0 |  |  |
| Number of Negative Values | | | | 0 | | 0 |  |  |
| Number of Missing Values | User-Missing | | | 0 | | 0 |  |  |
|  | System-Missing | | | 0 | | 0 |  |  |

**A**

**Linear**

| **Model Summary** | | | | | | | | | |  |  |  |  |  |  |
| --- | --- | --- | --- | --- | --- | --- | --- | --- | --- | --- | --- | --- | --- | --- | --- |
| R | R Square | | | Adjusted R Square | | | Std. Error of the Estimate | | |  |  |  |  |  |  |
| 1.000 | 1.000 | | | 1.000 | | | .000 | | |  |  |  |  |  |  |
| The independent variable is M. | | | | | | | | | |  |  |  |  |  |  |
| **ANOVA** | | | | | | | | | | | | | | |  |
|  | | | Sum of Squares | | | df | | Mean Square | | | F | | Sig. | |  |
| Regression | | | .000 | | | 1 | | .000 | | | 50226.235 | | .000 | |  |
| Residual | | | .000 | | | 8 | | .000 | | |  | |  | |  |
| Total | | | .000 | | | 9 | |  | | |  | |  | |  |
| The independent variable is M. | | | | | | | | | | | | | | |  |
| **Coefficients** | | | | | | | | | | | | | | | |
|  | | Unstandardized Coefficients | | | | | | | Standardized Coefficients | | | t | | Sig. | |
|  |  | B | | | Std. Error | | | | Beta | | |  |  |  |  |
| M | | .018 | | | .000 | | | | 1.000 | | | 224.112 | | .000 | |
| (Constant) | | .002 | | | .003 | | | |  | | | .507 | | .626 | |


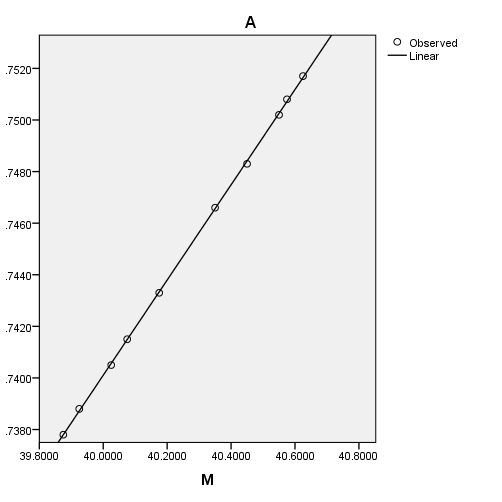


* Curve Estimation.

TSET NEWVAR=NONE.

CURVEFIT

/VARIABLES=A WITH M

/CONSTANT

/MODEL=LOGARITHMIC

/PRINT ANOVA

/PLOT FIT.

**Curve Fit**

| **Notes** | | | | | | | | |
| --- | --- | --- | --- | --- | --- | --- | --- | --- |
| Output Created | | | | | 11-DEC-2019 14:39:02 | | | |
| Comments | | | | |  | | | |
| Input | | Data | | | F:\fe.sav | | | |
|  |  | Active Dataset | | | DataSet1 | | | |
|  |  | Filter | | | <none> | | | |
|  |  | Weight | | | <none> | | | |
|  |  | Split File | | | <none> | | | |
|  |  | N of Rows in Working Data File | | | 10 | | | |
| Missing Value Handling | | Definition of Missing | | | User-defined missing values are treated as missing. | | | |
|  |  | Cases Used | | | Cases with a missing value in any variable are not used in the analysis. | | | |
| Syntax | | | | | CURVEFIT  /VARIABLES=A WITH M  /CONSTANT  /MODEL=LOGARITHMIC  /PRINT ANOVA  /PLOT FIT. | | | |
| Resources | | Processor Time | | | 00:00:00.16 | | | |
|  |  | Elapsed Time | | | 00:00:00.13 | | | |
| Use | | From | | | First observation | | | |
|  |  | To | | | Last observation | | | |
| Predict | | From | | | First Observation following the use period | | | |
|  |  | To | | | Last observation | | | |
| Time Series Settings (TSET) | | Amount of Output | | | PRINT = DEFAULT | | | |
|  |  | Saving New Variables | | | NEWVAR = NONE | | | |
|  |  | Maximum Number of Lags in Autocorrelation or Partial Autocorrelation Plots | | | MXAUTO = 16 | | | |
|  |  | Maximum Number of Lags Per Cross-Correlation Plots | | | MXCROSS = 7 | | | |
|  |  | Maximum Number of New Variables Generated Per Procedure | | | MXNEWVAR = 60 | | | |
|  |  | Maximum Number of New Cases Per Procedure | | | MXPREDICT = 1000 | | | |
|  |  | Treatment of User-Missing Values | | | MISSING = EXCLUDE | | | |
|  |  | Confidence Interval Percentage Value | | | CIN = 95 | | | |
|  |  | Tolerance for Entering Variables in Regression Equations | | | TOLER = .0001 | | | |
|  |  | Maximum Iterative Parameter Change | | | CNVERGE = .001 | | | |
|  |  | Method of Calculating Std. Errors for Autocorrelations | | | ACFSE = IND | | | |
|  |  | Length of Seasonal Period | | | Unspecified | | | |
|  |  | Variable Whose Values Label Observations in Plots | | | Unspecified | | | |
|  |  | Equations Include | | | CONSTANT | | | |
| **Model Description** | | | | | | | |  |
| Model Name | | | | | MOD_9 | | |  |
| Dependent Variable | | 1 | | | A | | |  |
| Equation | | 1 | | | Logarithmic | | |  |
| Independent Variable | | | | | M | | |  |
| Constant | | | | | Included | | |  |
| Variable Whose Values Label Observations in Plots | | | | | Unspecified | | |  |
| **Case Processing Summary** | | |  |  |  |  |  |  |
|  | N | |  |  |  |  |  |  |
| Total Cases | 10 | |  |  |  |  |  |  |
| Excluded Cases^a^ | 0 | |  |  |  |  |  |  |
| Forecasted Cases | 0 | |  |  |  |  |  |  |
| Newly Created Cases | 0 | |  |  |  |  |  |  |
| a. Cases with a missing value in any variable are excluded from the analysis. | | |  |  |  |  |  |  |
| **Variable Processing Summary** | | | | | | |  |  |
|  | | | | Variables | | |  |  |
|  |  |  |  | Dependent | | Independent |  |  |
|  |  |  |  | A | | M |  |  |
| Number of Positive Values | | | | 10 | | 10 |  |  |
| Number of Zeros | | | | 0 | | 0 |  |  |
| Number of Negative Values | | | | 0 | | 0 |  |  |
| Number of Missing Values | User-Missing | | | 0 | | 0 |  |  |
|  | System-Missing | | | 0 | | 0 |  |  |

**A**

**Logarithmic**

| **Model Summary** | | | | | | | | | |  |  |  |  |  |  |
| --- | --- | --- | --- | --- | --- | --- | --- | --- | --- | --- | --- | --- | --- | --- | --- |
| R | R Square | | | Adjusted R Square | | | Std. Error of the Estimate | | |  |  |  |  |  |  |
| 1.000 | 1.000 | | | 1.000 | | | .000 | | |  |  |  |  |  |  |
| The independent variable is M. | | | | | | | | | |  |  |  |  |  |  |
| **ANOVA** | | | | | | | | | | | | | | |  |
|  | | | Sum of Squares | | | df | | Mean Square | | | F | | Sig. | |  |
| Regression | | | .000 | | | 1 | | .000 | | | 42296.277 | | .000 | |  |
| Residual | | | .000 | | | 8 | | .000 | | |  | |  | |  |
| Total | | | .000 | | | 9 | |  | | |  | |  | |  |
| The independent variable is M. | | | | | | | | | | | | | | |  |
| **Coefficients** | | | | | | | | | | | | | | | |
|  | | Unstandardized Coefficients | | | | | | | Standardized Coefficients | | | t | | Sig. | |
|  |  | B | | | Std. Error | | | | Beta | | |  |  |  |  |
| ln(M) | | .743 | | | .004 | | | | 1.000 | | | 205.661 | | .000 | |
| (Constant) | | -2.001 | | | .013 | | | |  | | | -149.871 | | .000 | |


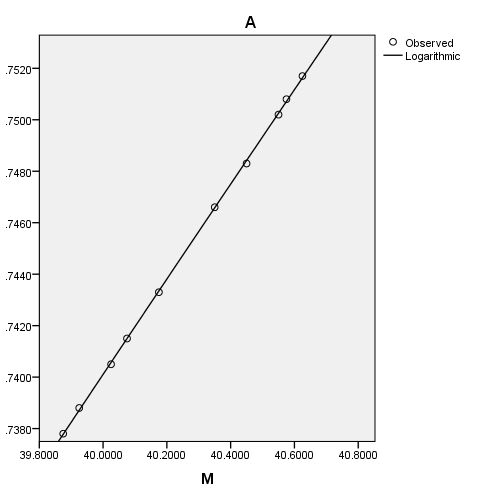


* Curve Estimation.

TSET NEWVAR=NONE.

CURVEFIT

/VARIABLES=A WITH M

/CONSTANT

/MODEL=INVERSE

/PRINT ANOVA

/PLOT FIT.

**Curve Fit**

| **Notes** | | | | | | | | |
| --- | --- | --- | --- | --- | --- | --- | --- | --- |
| Output Created | | | | | 11-DEC-2019 14:39:21 | | | |
| Comments | | | | |  | | | |
| Input | | Data | | | F:\fe.sav | | | |
|  |  | Active Dataset | | | DataSet1 | | | |
|  |  | Filter | | | <none> | | | |
|  |  | Weight | | | <none> | | | |
|  |  | Split File | | | <none> | | | |
|  |  | N of Rows in Working Data File | | | 10 | | | |
| Missing Value Handling | | Definition of Missing | | | User-defined missing values are treated as missing. | | | |
|  |  | Cases Used | | | Cases with a missing value in any variable are not used in the analysis. | | | |
| Syntax | | | | | CURVEFIT  /VARIABLES=A WITH M  /CONSTANT  /MODEL=INVERSE  /PRINT ANOVA  /PLOT FIT. | | | |
| Resources | | Processor Time | | | 00:00:00.14 | | | |
|  |  | Elapsed Time | | | 00:00:00.11 | | | |
| Use | | From | | | First observation | | | |
|  |  | To | | | Last observation | | | |
| Predict | | From | | | First Observation following the use period | | | |
|  |  | To | | | Last observation | | | |
| Time Series Settings (TSET) | | Amount of Output | | | PRINT = DEFAULT | | | |
|  |  | Saving New Variables | | | NEWVAR = NONE | | | |
|  |  | Maximum Number of Lags in Autocorrelation or Partial Autocorrelation Plots | | | MXAUTO = 16 | | | |
|  |  | Maximum Number of Lags Per Cross-Correlation Plots | | | MXCROSS = 7 | | | |
|  |  | Maximum Number of New Variables Generated Per Procedure | | | MXNEWVAR = 60 | | | |
|  |  | Maximum Number of New Cases Per Procedure | | | MXPREDICT = 1000 | | | |
|  |  | Treatment of User-Missing Values | | | MISSING = EXCLUDE | | | |
|  |  | Confidence Interval Percentage Value | | | CIN = 95 | | | |
|  |  | Tolerance for Entering Variables in Regression Equations | | | TOLER = .0001 | | | |
|  |  | Maximum Iterative Parameter Change | | | CNVERGE = .001 | | | |
|  |  | Method of Calculating Std. Errors for Autocorrelations | | | ACFSE = IND | | | |
|  |  | Length of Seasonal Period | | | Unspecified | | | |
|  |  | Variable Whose Values Label Observations in Plots | | | Unspecified | | | |
|  |  | Equations Include | | | CONSTANT | | | |
| **Model Description** | | | | | | | |  |
| Model Name | | | | | MOD_10 | | |  |
| Dependent Variable | | 1 | | | A | | |  |
| Equation | | 1 | | | Inverse | | |  |
| Independent Variable | | | | | M | | |  |
| Constant | | | | | Included | | |  |
| Variable Whose Values Label Observations in Plots | | | | | Unspecified | | |  |
| **Case Processing Summary** | | |  |  |  |  |  |  |
|  | N | |  |  |  |  |  |  |
| Total Cases | 10 | |  |  |  |  |  |  |
| Excluded Cases^a^ | 0 | |  |  |  |  |  |  |
| Forecasted Cases | 0 | |  |  |  |  |  |  |
| Newly Created Cases | 0 | |  |  |  |  |  |  |
| a. Cases with a missing value in any variable are excluded from the analysis. | | |  |  |  |  |  |  |
| **Variable Processing Summary** | | | | | | |  |  |
|  | | | | Variables | | |  |  |
|  |  |  |  | Dependent | | Independent |  |  |
|  |  |  |  | A | | M |  |  |
| Number of Positive Values | | | | 10 | | 10 |  |  |
| Number of Zeros | | | | 0 | | 0 |  |  |
| Number of Negative Values | | | | 0 | | 0 |  |  |
| Number of Missing Values | User-Missing | | | 0 | | 0 |  |  |
|  | System-Missing | | | 0 | | 0 |  |  |

**A**

**Inverse**

| **Model Summary** | | | | | | | | | |  |  |  |  |  |  |
| --- | --- | --- | --- | --- | --- | --- | --- | --- | --- | --- | --- | --- | --- | --- | --- |
| R | R Square | | | Adjusted R Square | | | Std. Error of the Estimate | | |  |  |  |  |  |  |
| 1.000 | 1.000 | | | 1.000 | | | .000 | | |  |  |  |  |  |  |
| The independent variable is M. | | | | | | | | | |  |  |  |  |  |  |
| **ANOVA** | | | | | | | | | | | | | | |  |
|  | | | Sum of Squares | | | df | | Mean Square | | | F | | Sig. | |  |
| Regression | | | .000 | | | 1 | | .000 | | | 34930.773 | | .000 | |  |
| Residual | | | .000 | | | 8 | | .000 | | |  | |  | |  |
| Total | | | .000 | | | 9 | |  | | |  | |  | |  |
| The independent variable is M. | | | | | | | | | | | | | | |  |
| **Coefficients** | | | | | | | | | | | | | | | |
|  | | Unstandardized Coefficients | | | | | | | Standardized Coefficients | | | t | | Sig. | |
|  |  | B | | | Std. Error | | | | Beta | | |  |  |  |  |
| 1 / M | | -29.914 | | | .160 | | | | -1.000 | | | -186.898 | | .000 | |
| (Constant) | | 1.488 | | | .004 | | | |  | | | 374.274 | | .000 | |


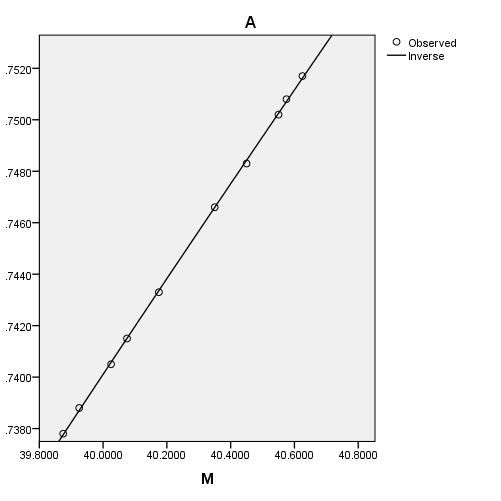

Supplement: Supplementary file 1 — Supplementary Information. [file 41598_2020_60657_MOESM1_ESM.docx]
